# Individual Nest Site Preferences Do Not Explain Upslope Population Shifts of a Secondary Cavity-Nesting Species

**DOI:** 10.3390/ani11082457

**Published:** 2021-08-21

**Authors:** Elisa J. Abeyta, Andrew W. Bartlow, Charles D. Hathcock, Jeanne M. Fair

**Affiliations:** 1 Environmental Stewardship, Los Alamos National Laboratory, Los Alamos, NM 87545, USA; hathcock@lanl.gov; 2 Biosecurity and Public Health, Los Alamos National Laboratory, Los Alamos, NM 87545, USA; abartlow@lanl.gov (A.W.B.); jmfair@lanl.gov (J.M.F.)

**Keywords:** behavioral plasticity, dispersal distance, elevation change, range shift, climate change, long-term monitoring, western bluebird

## Abstract

**Simple Summary:**

Environmental changes such as climate change have affected wildlife species behavior and geographic ranges globally. We analyzed nesting data of western bluebirds to determine whether the link between geographic range shifts of a western bluebird population in New Mexico, USA is due to individual adaptations or changes occurring at a larger scale. We looked at location data of marked and recaptured nestlings and adults that nested within our study area. We found that individual choices have no impact on the geographic range shift being observed in this population, suggesting that population-level processes, such as emigration and immigration, may be the main cause of these shifts.

**Abstract:**

Geographic ranges of plants and animals are shifting due to environmental change. While some species are shifting towards the poles and upslope in elevation, the processes leading to these patterns are not well known. We analyzed 22 years of western bluebird (*Sialia mexicana*) data from a large nest box network in northern New Mexico at elevations between 1860 m and 2750 m. This population has shifted to higher elevations over time, but whether this is due to changes in nesting behavior and preference for higher elevation within the population or driven by immigration is unclear. We banded adults and nestlings from nest boxes and examined nesting location and elevation for individual birds captured two or more times. Most recaptured birds nested at the same nest boxes in subsequent years, and the number of birds that moved upslope did not significantly differ from the number that moved downslope. Fledglings moved greater distances and elevations than adults, but these movements were not upslope specific. Female fledglings showed greater changes in elevation and distance compared to male fledglings, but again, movements were not consistently upslope. The upslope shift in this population may be due to birds immigrating into the population and not from changes in individual nesting behavior.

## 1. Introduction

Populations of plants and animals are responding to anthropogenic environmental change worldwide [1,2,3,4]. Climate change and changes to habitats mean that species must adapt to new environmental conditions [5] or shift their geographic ranges to match their preferred climate regimes (i.e., niche conservatism; [6]). Not all species have the ability to adapt quickly enough in the face of environmental change, making these populations more likely to undergo range shifts [7]. If a species can neither adapt nor shift its distribution because of geographic constraints, it may be vulnerable to local extinction [6,8]. This may be especially true for species that are range-restricted and for species that may already be pushed to the limits of their climatic or geographic niche; for example, species on mountaintops [9,10].

Climatic niches encompass the biotic and abiotic conditions where a species can persist over time and space [11] and are important to consider with the current rates of climate changes that are occurring worldwide. As climates change, niches also change, and a species must respond in order to persist. The effects of climate change and climate niches on geographic range shifts of populations and communities are still unpredictable, but general movement upslope and towards the poles has been observed in many species [12]. For example, Ref. [13] showed that the range of 14 out of 28 small mammal species in Yosemite National Park monitored for over a century significantly increased in elevation by an average of 500 m. Identifying whether range shifts occur through population-level processes, such as changes to reproductive success or dispersal (e.g., immigration and emigration), or through individual behaviors (e.g., changing nesting locations), can provide important information about which conservation efforts and mitigation tools should be implemented.

Despite geographic range shifts being documented more frequently, the processes behind these shifts have not been fully investigated. Bird species respond to environmental change in different ways; some adapt quickly through behavioral plasticity (e.g., timing of reproduction, resource use, microhabitat selection) or more slowly via evolution (i.e., genetic changes in the population). Species that do not adapt can contract, expand, or shift their geographic ranges [9,14,15,16,17,18]. Birds have been shown to move to higher latitudes and along elevational gradients; however, elevational shifts are not always predictable. Some species have shifted upslope, while others have shifted downslope [2,19,20,21,22].

Colonization and establishment events in new areas are critical for elevation shifts and can occur through two main processes [23,24]. First, individuals can shift their nesting preferences by using information about past breeding sites and choosing a new site the next year [24]. Thus, upslope and downslope population shifts occur when individual birds return to the same site to breed, but select and colonize higher or lower elevation nesting sites, respectively. Second, range shifts can occur as immigrants move into the area or fledglings disperse and nest near their natal area; but in doing so, they nest at different elevations because they are tracking preferred climatic conditions [23]. This kind of range shift occurs through population dispersal processes even though individual birds may nest in the same location year after year. Range shifts result from processes happening at the “leading edge” of a species distribution [15]. Studies suggest that range shifts could be due to pioneering individuals at the leading edge having different behaviors, morphology, or physiology that provide them the capacity to successfully expand beyond their typical range [25,26,27,28,29].

The western bluebird (*Sialia mexicana*) is a secondary cavity-nesting species in western North America that has a broad distribution up to 2900 m in elevation [30]. They are insectivorous, and the populations in and around our study area in northern New Mexico are residents or short-distance migrants. They are valuable indicators of ecosystem health because of their relatively quick responses to environmental change [31]. Throughout their range, males are the more philopatric sex and return to the natal site to breed or to help provision their parents’ nests, while females are the main dispersers [32,33]. Fledgling males either disperse to a new population to compete for a territory, or they inherit a territory from parents or relatives [28]. Over the past 19 years on the Pajarito Plateau, western bluebirds have not shifted the timing of their breeding even though spring temperatures have increased [22]. They have, however, shifted their nesting sites to higher elevations by an average increase of 5 m per year [22]. It is currently unknown if these elevational changes occurred through changes in individual behaviors of nesting preferences or through dispersal processes. Here, we analyze recapture data from 1997 to 2019 to understand changes in nesting elevation and dispersal distances in individual western bluebirds. Our data come from a nest box network that provides nesting sites for secondary cavity-nesting species, mostly occupied by bluebirds dispersed over an elevational range from 1860 m to 2750 m. The recapture data consist of adult birds that were captured two or more times and newly banded fledglings that have returned to the breeding site one or more times after fledging. Therefore, we were able to address the key question concerning individual nesting over time and test hypotheses regarding age and sex, both of which are important in nest site preference and postnatal dispersal. We tested the hypothesis that individual birds nest higher in elevation each consecutive year, thereby contributing to population shifts in elevation over time. Our null hypothesis was that individual birds do not nest higher in elevation each year, which would suggest that individual behavior is not the main process contributing to population shifts. We also tested hypotheses regarding differences between adults and fledglings and between male and female fledglings to document any differences in behavior. The first hypothesis was that fledglings will have greater changes in elevation and greater dispersal distances than adults, and that the average changes will be in the upslope direction. Regarding male and female fledglings, we compared elevational changes and dispersal distances from their natal areas to their new nesting sites. Because males are the philopatric sex, we hypothesized that female fledglings will show greater elevational changes and greater dispersal distances than male fledglings, and that these changes will tend towards upslope movement.

## 2. Materials and Methods

### 2.1. Study Location

This study was conducted at Los Alamos National Laboratory (LANL) and surrounding areas in Los Alamos, New Mexico, USA (35.09222° N, 106.3242° W, WGS84). The laboratory occupies ~103 km^2^ and is located on the Pajarito Plateau on the eastern flanks of the Jemez Mountains (see Musgrave et al. [34] for a recent map of nest box locations). The plateau is made up of narrow mesas, separated by steep-sided canyons. The primary habitats encompassed in the study area are predominantly pinyon-juniper forests and ponderosa pine (*Pinus ponderosa*) forests. Pinyon-juniper forests mainly comprise one-seed juniper (*Juniperus monosperma*) and pinyon pine (*Pinus edulis*) trees.

### 2.2. Field Work and Data Collection

In this study, we analyzed 22 years of data for western bluebirds using a network of nest boxes. We collected data from recaptured birds between the years 1997 and 2019. Western bluebirds were the main target species for the avian nest box network because they nest in secondary cavities. All of the nest boxes were located in pinyon-juniper or ponderosa pine forests between 1860 and 2750 m in elevation. Data collection for this network has been ongoing since 1997 using over 500 wooden nest boxes in 49 different sampling locations. Not all 49 sites were in operation simultaneously; a subset of these were monitored each year due to changes in staff over the years, inactive sites, or difficulty of accessing the nest boxes. A total of 350 to 550 boxes were monitored in any given year during this study. Commercial, standard-sized, wooden nest boxes that had a front-facing hinged door were used, allowing us the ability to collect data on the nest and its occupants. Nest boxes were placed ~50 m apart and mounted 2 m above ground.

During the breeding season, nest boxes in the network were monitored continuously by researchers. They were checked once every 2 weeks unless they were identified as being active. A nest was considered active if there was nesting material or eggs inside. When an active nest was identified, it was visited once every 5 days by researchers until nestlings hatched. The nest was visited again when nestlings were between the ages of 10–15 days old, so we could band each nestling. A last visit to the nest was to determine fledging success. A successful nest was an empty nest that had no evidence of predation, such as a flattened nest with an abundance of fecal matter within the box. A failed nest was determined by evidence of abandonment or predation, such as a box that was broken by a black bear (*Ursus americanus)*, missing eggs, or had cracked eggs. At this time, the nest box was cleaned out for a second clutch to be laid or for another pair to nest. Data collected on the nests included clutch size, hatch date, sex, and whether the nest successfully fledged young. Nestlings’ sex was determined by plumage color when they were 12 days or older, by the amount of blue on the wing and tail [32,35,36].

Many times, when a nest was active, females were captured in the nest box during incubation and banded. These opportunistic captures make up a large portion of our adult capture and recapture data. We recorded the sex and age and took morphometric measurements, including wing length, tail length, and mass. To obtain data on the parents that were not in nest boxes when they were visited, mist nets were set up near an active nest box to capture, band, and collect data on each adult. These adults were measured the same way as the individuals that were captured opportunistically. Data collectors acted in accordance with the Guidelines for the Use of Wild Birds in Research (Fair et al., 2010), and the approved Institutional Animal Care and Use Committee protocol. All New Mexico State and Federal Scientific Permits were obtained for all years of the project.

### 2.3. Statistical Analysis

We compiled all records of birds captured two or more times. This dataset contains the band numbers, dates of capture and recapture, nest box locations, ages, and sexes of adults and newly banded and fledged bluebirds (hereafter referred to as fledglings) between the years 1997 and 2019. These records were compiled to include the year, age, sex, location, and elevation of each nest box each year the bird was captured. For consistency, all fledglings that were banded in the nest were considered adults the next year(s) they were recaptured. Therefore, the adult category was made up of “after hatch year”’, “second year”, and “after second year” birds.

Elevation and distances between nest boxes (straight-line distances) were obtained using a geographic information system (Google Earth Pro 2020, version 7.3.3.7786, Google LLC, Mountain View, CA, USA). Data were visualized at the individual bird level for changes in elevation in subsequent years. To determine the positive, negative, and absolute changes in elevation from one year to the next, we used each bird movement or recapture as a data point. We used a linear model to test whether individual nesting increased in elevation over time (model residuals were normally distributed).

We used linear mixed models (LMM) and model selection to find the best variables that predicted both changes in distance and changes in elevation (absolute change in elevation). Our predictor variables were initial nesting elevation, year initially captured, sex, and age (fledgling/adult). We included initial nesting elevation to determine if where a bird initially nested predicted where it nested subsequently. Our random effect was bird ID. Year was not included as a random effect because it explained very little variation. We tested for multicollinearity using variance inflation factor (VIF) and found that all variables had values less than 3. We ran all combinations of models with an interaction between sex and age, and ranked the models using Akaike Information Criterion (AICc). Models with delta AICc values less than 2 were considered similar models in terms of predictability. Variables were considered significant if 95% confidence intervals did not cross zero. Models were run using the lme4 package [37], and model selection was performed using the MuMIn package [38].

We used chi-square tests to compare numbers of adults and fledglings and numbers of males and females to determine whether there were differences between groups in upslope and downslope shifts. Differences in elevation and distance between nest boxes were tested using Mann–Whitney U tests. Elevation and distance data were not normally distributed; and therefore, we opted to use non-parametric statistics. For those analyses for which birds were recaptured multiple times, we used a linear mixed model with bird ID as a random effect to test for these differences. All data analyses were completed in the statistical software program R (version 3.6.1 [39]). Data were visualized using the ggplot2 package [40].

## 3. Results

**Figure 1:**
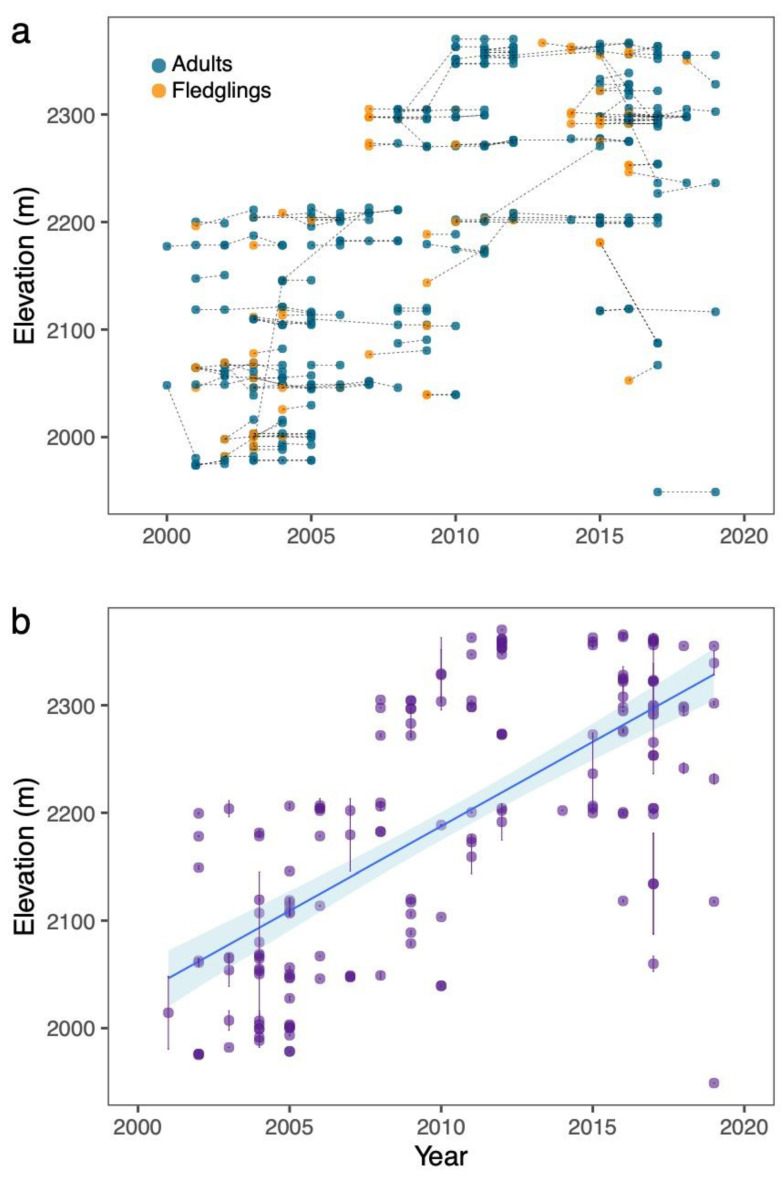
(**a**) The movement of nesting locations by elevation for individual birds year to year. Orange circles depict a bird that was banded as a nestling, and blue circles depict adults. Movements of an individual bird (*n* = 182) are tracked by dotted lines. (**b**) Linear regression of nesting elevation over time for recaptured birds (*n* = 182). Dots represent the mean (±SE) nesting elevation per bird. There was a significant positive relationship over time (LM: estimate = 15.69, SE ± 1.21, t = 12.93, *p* < 0.001, R^2^ = 0.48). Area shaded blue is the 95% confidence interval.

We recaptured a total of 182 individual bluebirds from 1997 to 2019, which consisted of 95 adults and 87 birds that were captured initially as fledglings. Of the 182 individuals, there were 148 birds that were recaptured only once. Twenty-nine birds were recaptured twice and 4 birds were recaptured three times. One individual was recaptured four times. These totals do not include same-year recaptures. These recaptures resulted in 222 data points (148 + 58 + 12 + 4) on potential individual bird movements (135 adults and 87 fledglings; Table 1). Each bird was plotted according to the year in which it was initially captured and then subsequently recaptured with the corresponding elevation of the nest box in which it nested for that year (Figure 1a). Birds that were first banded as fledglings were recorded at the location of their parents’ nests. These fledglings were considered adults the next year(s) they were captured.

Most individuals nested at approximately the same elevation year after year. However, a few birds changed elevation dramatically between subsequent nesting periods, both in the upslope and downslope directions (Figure 1a). From 2000 to 2019, birds generally nested at higher elevations later in the study period (i.e., 2011 to 2019) compared to the early part of the study period (i.e., 2000 to 2010). To test for an increase in elevation over time, we calculated the mean nesting elevation for each recaptured bird and plotted this against the last year each bird was captured (Figure 1b). All birds were adults because banded fledglings were considered adults when they were recaptured during a subsequent year. There was a significant positive relationship between year and elevation (LM: estimate = 15.69, SE ± 1.21, t = 12.93, *p* < 0.001, R^2^ = 0.48; Figure 1b).

For absolute change in elevation, the top linear mixed model included sex and age. This model was greater than 2 delta AICc units from the next model. In this model, age (adults: estimate [95% CIs] = −11.65 [−16.34 to −7.48]; SE = 1.99; df = 73.60; t = −5.87) and sex (females: estimate [95% CIs] = 10.98 [5.46 to 16.43]; SE = 2.76; df = 196.49; t = 3.97) were both significant. Fledglings and females had greater changes in elevation than adults and males, respectively. For change in distance, the top models included sex, age, and year. This model was greater than 2 delta AICc units from the next model. Age (age, adults: estimate [95% CIs] = −287.26 [−440.18 to −170.49]; SE = 54.48; df = 38.13; t = −5.27) and sex (sex, females: estimate [95% CIs] = 466.84 [236.54 to 694.14]; SE = 115.69; df = 184.68; t = 4.04) were both significant in this model, while year was not significant (estimate [95% CIs] = −6.69 [−27.04 to 13.76]; SE = 10.43; df = 163.99; t = −0.64).

**Figure 2:**
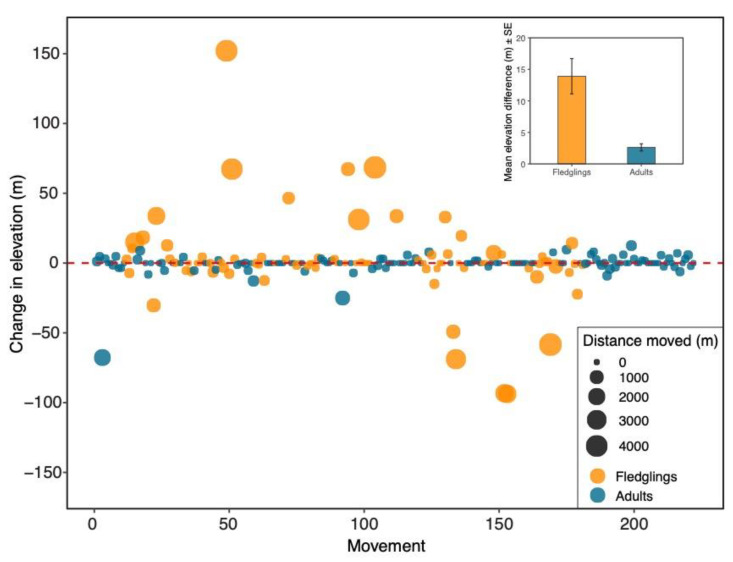
Age differences in elevational changes between nesting locations from one year to the next for each bird captured two or more times (*n* = 222). Each point represents an individual bird movement. An individual can have multiple points if it was captured more than two times. Orange circles depict a bird that was banded as a fledgling, and blue circles depict adults. The size of each point corresponds to the straight-line distance between nest boxes. Inset: Mean (±SE) elevation difference (absolute values) between fledglings and adults. Fledglings changed elevation more than adults (LMM: estimate = −8.48 ± 1.83, df = 90.97, t = −4.64, *p* < 0.001).

**Figure 3:**
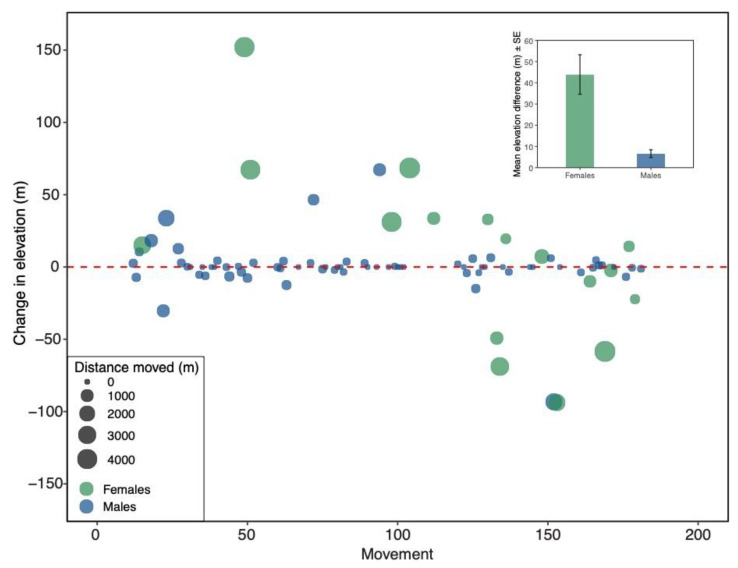
Sex differences in elevational changes between nesting locations from one year to the next for fledglings captured twice (*n* = 87). Each point represents an individual bird movement. Green circles depict females, and blue circles depict males. The size of each point corresponds to the straight-line distance between nest boxes. Inset: Mean (±SE) elevation difference (absolute values) between males and females. Females changed elevation more than males (Mann–Whitney U test: W = 719, *p* < 0.001).

We calculated the change in elevation, or lack of change, from the initial nest location to the subsequent nest location for adults and fledglings (Figure 2) and for male and female fledglings (Figure 3). The biggest upslope change in elevation for a bird between recapture years was 152.1 m, and the biggest downslope change in elevation was 93.8 m. Both of these were female fledglings. There were 68 upslope movements and 61 downslope movements (Table 1). There were 93 movements with no change in elevation, meaning they nested in the same nest box each capture (Table 1). There was no significant difference in the absolute elevation change between upslope movements and downslope movements (LMM: estimate = −1.18 ± 2.18, df = 23.61, t = −0.54, *p* = 0.60). The greatest distance moved between nest boxes was 4.5 km, by a female fledgling in the downslope direction.

The numbers of adult (*n* = 64) and fledgling (*n* = 65) movements that were upslope and downslope did not significantly differ (Chi-square test: X^2^ < 0.001, df = 1, *p* = 1.0; Table 1), meaning that adults and fledglings did not show patterns in how they were moving between boxes. However, when numbers of adults and fledglings that did not move were included, there was a significant difference between adults and fledglings (Chi-square test: X^2^ = 16.21, df = 2, *p* < 0.001; Table 1). Specifically, more adults remained at the same nest box than fledglings (71 adults vs. 22 fledglings). There was a significant difference in the absolute change in elevation between adults and fledglings (LMM: estimate = −8.48 ± 1.83, df = 90.97, t = −4.64, *p* < 0.001; Figure 2 inset); fledglings changed elevation more than adults. In those fledglings that moved to different nest boxes, there was no significant difference between upslope elevation changes and downslope elevation changes (Mann–Whitney U: W = 579, *p* = 0.50). Likewise, there was no significant difference between upslope and downslope elevation changes for adults (LMM: estimate = 0.14 ± 1.59, df = 11.57, t = 0.086, *p* = 0.93), meaning that there were no patterns in elevation shifts for individual birds. Adults and fledglings differed significantly in the straight-line distance traveled between nest boxes (LMM: estimate = −218.61 ± 50.72, df = 50.45, t = −4.31, *p* < 0.001; Figure 2); fledglings moved significantly greater distances than adults.

The changes in elevation between male and female fledglings were also compared (Figure 3). Out of the 87 fledglings that returned to breed in the same general area, 70 (80.4%) were male. There was not a significant difference between the numbers of male and female fledglings that shifted upslope and downslope (Chi-square test: X^2^ = 0.118, df = 1, *p* = 0.73; Table 1). However, when numbers of males and females that did not move were included, there were significantly more males (*n* = 22) than females (*n* = 0) that did not move (Chi-square test: X^2^ = 7.63, df = 2, *p* = 0.02; Table 1). There was a significant difference between fledgling male and female absolute changes in elevation (Mann–Whitney U test: W = 719, *p* < 0.001; Figure 3 inset); females changed elevation more than males. Male and female fledglings differed significantly in the straight-line distance traveled between nest boxes (Mann–Whitney U test: W = 772, *p* < 0.001), such that females dispersed farther than males (Figure 3).

Some of the fledglings did not change elevation and were even captured from the same nest box in which they were born. Twenty-two male fledglings came back to the same nest the following year. In addition, out of the 34 birds captured more than two times, 14 were initially captured as fledglings. Of these 14 fledglings, 12 (87.5%) were male, the philopatric sex.

## 4. Discussion

Environmental changes are causing birds to change behaviors, adapt to these changes, or shift their geographic ranges [20,21,24]. Geographical range shifts can be due to individual movements each year or through colonization and establishment events at the leading edge of a species distribution. The main goal of this study was to understand elevational changes and dispersal distances of individual western bluebird adults and fledglings in subsequent years. Knowing how species are responding to environmental changes will help predict behavioral responses and aid in management practices in the future.

We found no patterns in individual changes from year to year. The number of individual movements in the downslope direction was similar to that in the upslope direction. Likewise, the magnitude of change in elevation was the same in the downslope direction as the upslope direction. Most birds nested in the same nest box year after year, especially adult birds. This is contrary to our first hypothesis that individual birds nest at higher elevations each year and that these changes contribute to population shifts over time. We saw a general upslope pattern in nesting over time (Figure 1b), which is the same pattern documented in the same population in Wysner et al. [22] and consistent with other patterns in birds globally [15,19,41,42]. Our results suggest that this shift over 22 years was not caused by consistent changes in individuals’ nesting preferences year to year.

We cannot reject our second hypothesis, which was that immigrating individuals are tracking preferred climate conditions and nesting higher, on average, than the current population. Although we have no data regarding the proportion of birds immigrating to the area, this scenario seems likely for three reasons. First, adult birds that re-nest and breed the following year generally nest in the same general location (i.e., at the same elevation). Second, fledglings that returned to the same area to breed showed greater elevation changes, and distances traveled, the following year than adults. However, these fledglings showed similar elevational changes upslope as downslope. This is also contrary to our hypothesis that fledgling movements would be consistently upslope. Third, site fidelity of fledglings is fairly low in our population, suggesting that the majority of birds have immigrated from other populations.

Pioneering individuals have been shown to have different traits than other members of the population for birds, reptiles, amphibians, and mammals [25,43,44,45]. Morphological, behavioral, and physiological traits may predispose certain individuals to disperse farther [25,26,27]. It is currently unclear if bluebirds that nest higher in our population have different traits that make them predisposed to disperse farther, especially upslope into the leading edge of population expansion. In an expanding population of western bluebirds in the northwestern United States, dispersal is biased toward highly aggressive males [28,29]. Western bluebird fledglings can acquire nest sites and territories from parents and relatives, or they can disperse to a new population and compete for their own territory [28]. Based on our data, it seems that fledglings are staying near their parents’ nest and territory rather than dispersing upslope to the leading edge of the population. Bluebirds that immigrate to the population and nest at higher elevations may be more aggressive dispersers or are simply tracking local climatic conditions upon arrival.

The fledglings that returned to breed were mostly males (80.4%). Male fledglings moved significantly less in elevation and distance the year after they fledged compared to female fledglings. This is similar to other studies that have shown male western bluebirds to be philopatric to their natal site [32,35,46], and is consistent with our hypothesis. Males are known to return to their natal site to help their parents raise young, and can even help their parents with a second nest in the same season [33,35,47]. By helping, these helper males increase their, and their parents’, inclusive fitness [48]. Although, females showed greater elevational changes than males, the magnitude of changes was the same in the upslope and downslope direction, contrary to our hypothesis. A future question to address would be whether males and females differ in their immigration rates, and whether there are sex differences in pioneer individuals at the leading edge of range shifts.

Our sample size was relatively small despite the long timespan for which we collected data. We have banded 6597 bluebirds since this nest box network was initiated, and have a recapture rate of 3.4%. However, this is based on actively mist-netting adults near nest boxes as well as opportunistic recaptures (i.e., grabbing adults off the nest). Future work will involve tracking nestlings using radio transmitters to compare male and female dispersal and determine where fledglings disperse to. Fledglings, particularly males, may come back to their parents’ nest to help and may also have their own nest. However, the majority of fledglings disappear from our study area and may disperse into new populations. In this population, there is generally high fledging success, even when parasitized by nest parasites [34]. After fledging, predation is a large risk factor for fledglings and may also contribute to why we may see less recaptures of fledglings [49].

## 5. Conclusions

Changes to plant and animal distributions are difficult to predict because behavioral, morphological, and physiological traits vary greatly between individual species. We found that the western bluebirds in this study did not significantly move upslope or downslope in elevation year to year in response to environmental change. This suggests that geographic range shifts are not occurring at the individual level, but rather through immigrants nesting at higher elevations, although more research is needed to explicitly test this hypothesis. Future work should focus on understanding the traits of bluebirds at the upslope edge of the range compared to the rest of the population on the Pajarito Plateau [50]. This would provide more information regarding potential mechanisms for species range shifts, as predictions on a per species basis still remain a challenge.

## Figures and Tables

**Table 1:** The numbers of movements that were upslope, downslope, and showed no change for adults and fledglings. Both adults and fledglings have been split into males and females.

	Number of Upslope Shifts	Number of Downslope Shifts	Number Showing No Change	Total
Adults	34	30	71	135
*Males*	14	9	21	44
*Females*	20	21	50	91
Fledglings	34	31	22	87
*Males*	24	24	22	70
*Females*	10	7	0	17
Total	68	61	93	222

## Data Availability

The datasets generated during and/or analyzed during the current study are available from the corresponding author on reasonable request.
